# Impact of Publicly Financed Health Insurance Schemes on Healthcare Utilization and Financial Risk Protection in India: A Systematic Review

**DOI:** 10.1371/journal.pone.0170996

**Published:** 2017-02-02

**Authors:** Shankar Prinja, Akashdeep Singh Chauhan, Anup Karan, Gunjeet Kaur, Rajesh Kumar

**Affiliations:** 1 School of Public Health, Post Graduate Institute of Medical Education and Research, Chandigarh, India; 2 Indian Institute of Public Health, Delhi, Public Health Foundation of India, Delhi NCR, India; Tianjin University of Technology, CHINA

## Abstract

Several publicly financed health insurance schemes have been launched in India with the aim of providing universalizing health coverage (UHC). In this paper, we report the impact of publicly financed health insurance schemes on health service utilization, out-of-pocket (OOP) expenditure, financial risk protection and health status. Empirical research studies focussing on the impact or evaluation of publicly financed health insurance schemes in India were searched on PubMed, Google scholar, Ovid, Scopus, Embase and relevant websites. The studies were selected based on two stage screening PRISMA guidelines in which two researchers independently assessed the suitability and quality of the studies. The studies included in the review were divided into two groups i.e., with and without a comparison group. To assess the impact on utilization, OOP expenditure and health indicators, only the studies with a comparison group were reviewed. Out of 1265 articles screened after initial search, 43 studies were found eligible and reviewed in full text, finally yielding 14 studies which had a comparator group in their evaluation design. All the studies (n-7) focussing on utilization showed a positive effect in terms of increase in the consumption of health services with introduction of health insurance. About 70% studies (n-5) studies with a strong design and assessing financial risk protection showed no impact in reduction of OOP expenditures, while remaining 30% of evaluations (n-2), which particularly evaluated state sponsored health insurance schemes, reported a decline in OOP expenditure among the enrolled households. One study which evaluated impact on health outcome showed reduction in mortality among enrolled as compared to non-enrolled households, from conditions covered by the insurance scheme. While utilization of healthcare did improve among those enrolled in the scheme, there is no clear evidence yet to suggest that these have resulted in reduced OOP expenditures or higher financial risk protection.

## Introduction

Achieving Universal Health Coverage (UHC) is a major policy goal in health sector globally. [1, 2] Despite the acceptance of UHC at policy level in India, around three-quarters of healthcare spending is borne by households. [3] The recent National Sample Survey (NSS) report reveals that only 12% of the urban and 13% of the rural population is under any kind of health protection coverage. [4] Not surprisingly, nearly 26% of the total health spending by rural households is sourced from either borrowings or selling of assets. [4] Further, OOP spending pushes approximately 3.5% to 6.2% of the India’s population below the poverty line every year. [5–7]

Traditionally, health care financing in India had been mostly restricted to the supply side, focussing on the strengthening of infrastructure and human resource. The advent of National Rural Health Mission (NRHM) in 2005 also served as an instrument of strengthening the supply-side infrastructure. [8] The Planning Commission’s High Level Expert Group (HLEG) proposed a model to achieve UHC under which citizens would have full access to free healthcare from a combination of public and private facilities. [9] This shifted government’s attention from its prior focus on supply side to demand side financing models in the form of publicly sponsored health insurance schemes.

Since 2007, several publicly financed health insurance schemes have been launched in India both at the state level such as *Rajiv Aarogyasri* Health Insurance Scheme (RAS) in Andhra Pradesh [10], *Rajiv Gandhi Jeevandayee Arogya Yojana* (RGJAY) in Maharashtra [11], Chief Minister’s Comprehensive Health Insurance scheme (CMCHIS) in Tamil Nadu [12], and *Rashtriya Swasthya Bima Yojana* (RSBY) at the Central level. [13] These demand-side financing mechanisms entitle poor and other vulnerable households to choose cashless healthcare from a pool of empanelled private or public providers. While the RSBY scheme was designed and implemented by the Ministry of Labour and Employment (MOLE), the implementation role for RSBY–now called *Rashtriya Swasthya Suraksha Yojana* (RSSY, however we refer to as RSBY in the entire paper), has been recently transferred to Ministry of Health and Family Welfare in 2015. [14]

In the last 7–8 years, a large amount of government’s money has been invested in the implementation of these health insurance schemes. A total of INR 370 billion (USD 587 million) tax money has been allocated for RSBY since its launch in 2008–09. [15] If the budgets of state sponsored schemes are also pooled, it amounts to a significant amount of public exchequer’s money, thereby justifying a need to determine whether these schemes are achieving their desired objectives.

In line with this policy need for an appraisal, the Government of India constituted a task force on costing of health services. One of the terms of reference for this Task Force included an assessment of RSBY. [16] Also, several State Governments have set up independent commissions to determine the best way forward to achieve universal health coverage. [17, 18] As a result, there is a need to systematically review evidence in terms of whether these schemes have been able to achieve the objectives of universalizing health care for which they were launched. Two reviews have been published earlier, both of which measured the impact of health insurance in low and middle income countries as a whole without a specific focus on India. [19, 20] Specific characteristics of the scheme implementation and contextual differences in various countries support a case for a systematic review with a national focus. Further, one of these review focussed on only social and community based health insurance schemes. [20] However, much of the current interest is on determining success or failure of tax-funded health insurance schemes which cover nearly 14% out of the total 15% population who have any form of health care insurance.

As a result, we conducted a systematic review to primarily assess the impact of publicly financed health insurance schemes on utilization of health care services, out of pocket expenditure, financial risk protection and on the health of population in India. Secondly, we also summarise the findings of various process evaluations, which have assessed the performance of these schemes in terms of extent of community awareness, determinants of enrolment and utilization, accessibility and utilization of different services across states in India.

## Methodology

### Search strategy

A comprehensive computerised search was conducted to search for empirical studies focussing on the impact or evaluation of publicly sponsored health insurance schemes in India. PubMed, Google scholar, Ovid, Scopus and Embase databases were searched to identify eligible studies published till September 2015. Official websites of various health insurance schemes (www.rsby.gov.in, www.aarogyasri.telangana.gov.in, www.sast.gov.in/home/VAS.html, http://www.cmchistn.com and /www.chiak.org) were also searched. The review used the search strategy consisting of following key words:

(((((((((((Publicly sponsored health insurance) OR government sponsored health insurance) OR Rashtriya Swasthya Bima Yojana) OR RSBY) OR rajiv arogyasree health insurance scheme) OR rajiv aarogyasri community health insurance scheme) OR vajpayee arogyasri) OR vajpayee arogyasri yojana) OR chief minister kalaignar insurance scheme) OR rajiv gandhi jeevandayee arogya yojana) OR comprehensive health insurance scheme)”.

The search strategy was defined by reviewing the previously done systematic reviews and in consultation with the research staff from the Advanced Centre for Evidence-Based Child Health and the library staff of the Post-Graduate Institute of Medical Education and Research, Chandigarh. The key words were checked for controlled vocabulary under Medical Subject Headings (MeSH) of PubMed. Two investigators (ASC and GK) had access to abstract and full text of the paper to decide on its inclusion. Discrepancies between the two investigators were solved by discussion with the third investigator (SP). Two authors of this review are familiar with the methods of systematic review (SP and AK), two are experts in health economics with strong interest and familiarity with the health financing policies (SP and AK), while another author is a senior public health expert (RK).

### Inclusion criteria and study selection

The review included peer-reviewed articles, government reports and working papers that were reported in the English language and excludes abstracts, expert opinions, narrative reviews, commentaries, case reports and conference papers.

**Fig 1:**
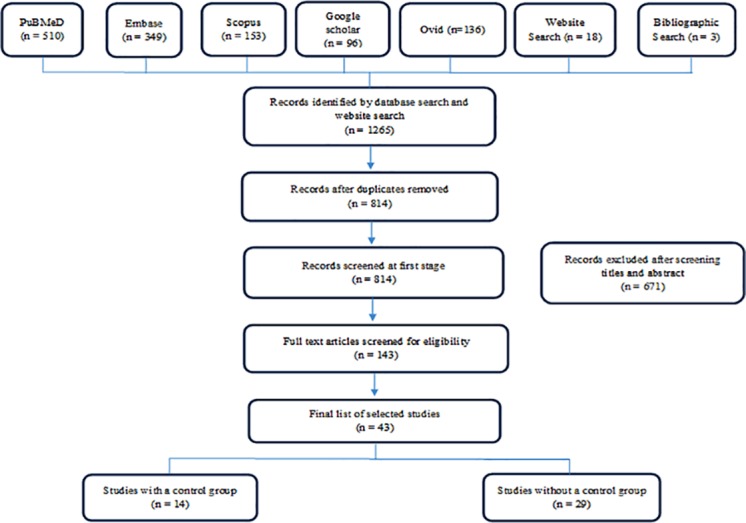
Flowchart showing selection of studies.

The studies were selected based on a two stage screening process as per PRISMA guidelines [21] (S1 Table). The first step comprised of searching for studies based on the search strategy from the selected databases and websites. Following this, duplicates were removed and the remaining studies were then screened by applying inclusion criteria to the titles and abstracts. Based on the screening of titles and abstracts, potentially relevant articles were selected for further review, which involved examining the content of their full text. After reviewing full text, only empirical research studies were considered eligible while others were excluded. At this stage, a bibliographic search of the selected studies was also carried out to identify additional relevant articles. The search was continued until no new article was found (Fig 1).

### Data extraction and quality

A standardised data extraction form was developed to collect information from the selected studies on the relevant impact outcomes, besides the general and methodological aspects. The latter included information on year of publication, funding agency, study design or type of study (experimental and observational), description of intervention and control group, duration and location of the study, sample size, type of outcome assessed, etc. Two researchers (ASC and GK) independently extracted the data and assessed the quality of the studies.

The studies selected in the review were divided into two groups i.e., with a comparison or control group (against which the insured group was measured) and without a control group (descriptive in nature). To assess the impact on utilization, OOP expenditure and health indicators, studies with a comparison group alone were reviewed. Process level indicators were assessed based on the findings of studies from both the groups, i.e. with and without control group. Further, quality of these studies was assessed by Effective Public Health Practice Project (EPHPP) quality assessment tool for quantitative studies. [22] The components of quality assessment in the EPHPP tool include type of study, presence of any kind of selection bias, consideration to blinding and confounders, validity and reliability of the data collection tools and consideration to withdrawals and loss to follow ups, if any. We also categorised the studies (having a control group) based on their analytical approach–i.e. Intention to Treat (ITT) and Average Treatment effect on the Treated (ATT) analysis. [23] Basically, ITT measures impact on the eligible population irrespective of getting enrolled or utilising the services while ATT measures impact on those who are enrolled in the scheme.

## Results

A total of 1265 articles were identified from databases (n = 1244), websites (n = 18) and bibliographic search (n = 3) as shown in Fig 1. After removing duplicates, the remaining 814 articles were screened by applying inclusion criteria to the titles and abstracts. A total of 671 articles were excluded in the 1^st^ stage screening and 143 studies were identified as eligible for 2^nd^ screening. Full text papers of these 143 studies were reviewed. Ultimately, 43 articles were found eligible for this systematic review. Out of this, 14 studies had a comparison group [24–37] and the remaining 29 were without a comparison group [38–66].

### General characteristics of selected studies

Out of the 14 studies with a comparison group, 7 were cross-sectional studies with data collected from intervention and control group, while 6 studies were quasi experimental in nature adopting a pre and post design. Out of these 6 studies, 2 studies evaluated the impact based on difference in difference analysis and one study followed geographic discontinuity design (Table 1). Most of these studies (n = 8) were published in peer reviewed journals while the remaining were reports (n = 3) and working papers (n = 3). Around half of the studies (n = 6) evaluated RSBY scheme, followed by studies on RAS (n = 3), *Vajpayee Aarogyashri* Scheme (VAS) (n = 1) and Comprehensive Health Insurance Scheme in Kerala (n = 1). Further, focus of the remaining 3 studies was on both RSBY and RAS. Twelve studies evaluated the health insurance scheme within 3 years of their implementation while the remaining 2 studies evaluated the scheme following 3 years of implementation.

**Table 1:** Characteristics of the selected studies.

Characteristics	Number of Studies	
	With a comparison group	Without a comparison group
**Study design**		
Cross sectional	7	29
Pre and post design	4	0
Difference in difference design based on before and after implementation of the scheme	2	0
Geographic discontinuity design	1	0
**Duration between implementation of the scheme and evaluation of the study**		
Equal to or less than 3 years	12	16
Greater than 3 years	2	9
Not clear	0	4
**Type of publication**		
Peer reviewed journal	8	17
Government reports	3	4
Working papers	3	8
**Type of scheme**		
RSBY	6	24
Rajeev Aarogyashree Scheme	3	3
Vajpayee Aarogyashree Scheme (Karnataka)	1	0
Comprehensive Health Insurance Scheme (Kerala)	1	0
Chief Minister Health Insurance Scheme, Tamil Nadu	0	1
RSBY and Rajeev Aarogyashree Scheme	3	0
RSBY and Vajpayee Aarogyashree Scheme	0	1
**Impact outcome**		
Utilization	6	0
Financial risk	13	0
Health indicator	1	0
**Geographic focus of the study**		
Maharashtra	2	3
Uttar Pradesh	1	0
Karnataka	2	2
Kerala	2	1
Andhra Pradesh	2	3
Chhattisgarh	0	5
Delhi	0	2
Gujarat	0	3
Himachal Pradesh	0	1
Tamil Nadu	0	1
Maharashtra and AP	3	0
Bihar, Uttrakhand and Karnataka	1	0
More than 5 Indian states	1	8
**Year of Publication**		
2009	1	0
2010	1	3
2011	1	6
2012	3	4
2013	1	11
2014	5	2
2015	2	1
Not stated	0	2
Total	14	29

With regards to studies without a comparison group (n = 29), majority of them (59%, n = 17) were published in peer reviewed journals, 28% (n = 8) were working papers and the remaining were reports (13%) (Table 1). All the studies had a cross sectional study design, out of which 8 studies were based on secondary data and 4 had a regression model based analysis. Nearly 83% (n = 24) of the studies evaluated RSBY, followed by 10% studies (n = 3) on RAS. More than half (56%, n = 16) of these studies were done within 3 years of the implementation of the scheme, followed by 31% (n = 9), assessing the scheme following 3 years of implementation. For the rest, 13% of the studies duration between implementation of the scheme and evaluation of the study was not clearly stated in the article.

### Impact assessment

Table 2 summarises the impact of various publicly financed health insurance schemes reported in the selected 14 studies with a comparison group. Nine of these studies were based on ATT analysis approach [26–29, 31, 34–37], while remaining 5 studies were ITT in nature. [24, 25, 30, 32, 33]

**Table 2:** Methodological characteristics and findings of the studies with a comparison group.

Study Author &Year	Study design	Source of data and Methodology	Time period after implementation of the scheme	Quality of the study	Impact on Utilization	Impact on Financial risk protection*	Health impact
**Rao et al., 2014 [24]**	Quasi experimental design (Pre and post design)	Primary survey in the states Andhra Pradesh and Maharashtra and comparison with the findings of NSS^@^ 2004–05 round.	3 years	Strong	Utilization increased in both states; more increase in Andhra Pradesh than Maharashtra	Inpatient expenditure, large expenditure (proxy for catastrophic health expenditure) increased over the time period with more increase in Maharashtra than Andhra Pradesh.	
**Selvaraj et al., 2012 [25]**	Quasi experimental design (Pre and post design)	National Sample Survey rounds for the year 2004–05 and 2009–10 were compared	< 3 years	Moderate		OOP^$^ inpatient spending, catastrophic headcount ratio and OOP spending as a proportion of overall spending increased over the time.	
**Amicus Advisory Pvt. Ltd. [29]**	Cross sectional	Primary survey in 10 villages of Jaunpur district, Uttar Pradesh	< 3 years	Weak		Eligible and users of the scheme incurred less expenditure than non-users.	
**Aiyar et al., 2013 [26]**	Cross sectional	Two rounds of data collection from the 2 districts of Karnataka, 2 years apart.	< 3 years	Weak	Incidence of hospitalization increased among insured than non-insured.	OOP expenditure and catastrophic health expenditure increased in both insured and non-insured households.	
**Sunny et al. [27]**	Cross sectional	Primary data collected from the insured and non-insured hospitalised cases in the state of Kerala.	< 3 years	Moderate		There was similar amount of expenditure incurred by both insured and non-insured cases.	
**GIZ, 2012 [28]**	Cross sectional	Primary survey conducted across three states of Bihar, Uttrakhand and Karnataka.	>3 years	Moderate		90% of the insured households did not spend any money on hospitalization.	
**Fan et al., 2012 [33]**	Quasi experimental design (Pre and post design with a DID* based analysis)	National Sample Survey rounds for the year 1999–2000, 2004–05 and 2007–08 were compared.	< 3 years	Strong		Initial reduction in OOP expenditure and catastrophic health expenditure, followed by an increase in inpatient expenditure.	
**Bergkvist et al., 2014 [30]**	Quasi experimental design (Pre and post design with a DID based analysis)	Primary survey in the states of Andhra Pradesh and Maharashtra; results compared with NSS 2004–05 round.	3 years	Strong	Increased rate of utilization, with faster increase among both the poor and the better off in Andhra Pradesh than Maharashtra.	Smaller growth in OOP expenditure in Andhra Pradesh compared to Maharashtra and mainly concentrated among the richest 60%.	
**Dhanaraj et al., 2014 [31]**	Quasi experimental design (Pre and post with analysis based on panel logit model)	Panel longitudinal dataset of Young Lives project of rounds 2002, 2006, and 2009 was compared for the state of Andhra Pradesh.	< 3 years	Strong		No significant effect in reduction of OOP expenditure over the time period.	
**Katyal et al., 2015 [32]**	Quasi experimental design (Pre and post design with a DID based analysis)	A primary survey undertaken in the states Andhra Pradesh and Maharashtra and the results was compared with the findings of NSS 2004–05 round.	3 years	Strong	Utilization of private hospitals increased in Andhra Pradesh and decreased in Maharashtra. Utilization of public facilities declined in both the states with more decline in Andhra Pradesh.	OOP increased both in public and private facilities, with greater increase in Maharashtra than Andhra Pradesh.	
**Mitchell et al., 2011 [34]**	Cross sectional	Primary household survey conducted in two districts of Andhra Pradesh	< 3 years	Weak		Households with insurance reported higher OOP expenses than those without insurance.	
**Philip et al., 2012 [35]**	Cross sectional	Primary survey conducted in the state of Tamil Nadu.	< 3 years	Strong	Utilization was significantly high among insured as compared to non-insured.	Mean OOP expenses among insured was significantly higher than uninsured households.	
**Sood et al., 2014 [36]**	Quasi experimental design (Geographic discontinuity design with analysis based on logit model)	Primary surveys conducted between the communities where scheme has and has not been implemented in the state of Karnataka	< 3 years	Strong	Insured households were more likely to use the facilities as compared to non-insured.	There was reduction in OOP expenditures among insured as compared to non-insured families.	Enrolled households had relatively lower mortality rate from conditions covered by the scheme.
**Ghosh et al., 2014 [37]**	Cross sectional	Primary survey conducted in the state of Maharashtra.	5 years	Moderate	Utilization was higher among the insured than non-insured families.		

* DID: difference in difference

^@^ NSS: national sample survey

^$^ OOP: out-of-pocket.

Among these, 7 studies (50%) assessed financial risk protection only, one study measured utilization alone, while remaining 5 studies (36%) evaluated both utilization and financial risk protection. Only one study included all the impact outcomes including the impact of insurance on the health of the population.

#### Financial risk protection

Out of the 13 studies assessing financial risk protection [24–36], 9 (69%) reported no reduction in OOP expenditure among enrolled households after implementation of health insurance schemes. [24–27, 30–32, 34, 35] In terms of quality, 7 studies had a strong methodological design [24, 30–33, 35, 36], out of which 5 reported increase in the OOP expenses. [24, 30–32, 35] The remaining 2 studies, which evaluated state sponsored insurance schemes of Andhra Pradesh and Karnataka, showed a decline in OOP expenses. [33, 36] Out of the five strong quality studies showing increase in OOP expenditure, 3 studies were based on the same data and methodology but had measured varied outcomes in terms of financial protection. [24, 30, 32] Specifically, among studies measuring catastrophic health expenditure as a measure of financial protection, 3/4^th^ showed increase in the incidence of catastrophic health count. [24–26] Only a single high quality study, which evaluated Andhra Pradesh’s RAS scheme showed a reduction in incidence catastrophic head count after implementation of the scheme. [33]

The studies (n = 5) which measured the impact of RSBY only, were either of a low or moderate quality and among these, 2 studies reported a reduction in OOP expenses [28, 29], but none showed any decrease in incidence of catastrophic health expenditure. Among the 4 studies which evaluated state sponsored schemes [33–36], 2 reported reduction in OOP expenses [33, 36], and one study showed decrease in number of catastrophic head count [33]. One study which considered all the publicly sponsored health insurance schemes together as one, reported that all these were associated with rise in OOP expenditure and catastrophic health expenditure. [25]

Three studies, which were based on similar data and methodology, compared the impact of RAS in Andhra Pradesh with that of RSBY in Maharashtra. [24, 30, 32] One of these studies showed that in both the states, schemes were associated with increase in OOP expenditure and catastrophic health expenditure, with higher increase in the state of Maharashtra. [24] Other study showed that this increase in expenditure was observed among both the household groups who accessed care in public or private health facilities. [32] The latter finding implied some protective effect of RAS in Andhra Pradesh, relative to RSBY in Maharashtra. However, independently, RAS did not result in a reduction in OOP expenses among insured. Another study inferred that this relative reduction in OOP expenditure and catastrophic health expenditure in Andhra Pradesh (compared to Maharashtra) was concentrated more among the richest 60%, implying an inequitable effect. [30]

Among 7 studies with a quasi-experimental design, 5 showed that the insurance schemes were associated with a rise in OOP expenditure. [24, 30–32, 35] Similarly, among the 3 studies based on DID analysis, 2 reported showed rise in OOP expenditure. [24, 25] Among 6 cross sectional studies, a study reported similar [27] amount of OOP expenditures among enrolled and non-enrolled group and 2 studies reported reduction in incurring of OOP expenses. [28, 29]

Out of the 7 studies with a strong methodological design, 4 were done within 3 years of the implementation of the schemes, of which 2 studies reported reduction in OOP expenditure [33, 36] and a study showed reduction in catastrophic health expenditure. [33] Studies done at and after 3 year of implementation showed, that schemes were associated with increase in OOP expenses and number of catastrophic head count. [24, 28, 30, 32]

#### Utilization

Overall 7 articles assessed the impact of health insurance on utilization of health services and the findings of all these studies showed that these insurance schemes were associated with increase in consumption of health care services. In terms of quality, 5 studies were of strong methodological rigour [24, 30, 32, 35, 36] and the remaining 2 had a moderate or weak quality. [26, 37] The increase in utilization among these studies varied from 12.3% to 244% among the insured as compared to non-insured households. The studies based on ATT analysis showed that this increase was in in the range of 12.3%-244%, [26, 35–37] whereas studies based on ITT analysis showed the increase in the range of 22%-56% among the enrolled households. [24]

Among the studies which evaluated RSBY alone (n = 2), increase in utilization varied from 15.3% in Maharashtra [37] to 244% in Karnataka. [26] For the state-specific insurance schemes, increase in consumption of health care varied from 12.3% in Karnataka’s VAS [36] to 35.4% for Comprehensive Health Insurance Scheme of Kerala. [35]

One out of the 3 studies which were based on same data and methodology, comparing the impact of RAS in Andhra Pradesh with that of RSBY in Maharashtra, showed an increase in utilization in post insurance period in both states with higher increase in the state of Andhra Pradesh. [24] Another study showed that this significant positive growth in the utilization was more among both the poor and better-off households in Andhra Pradesh as compared to Maharashtra. Further, it also showed that the increase in utilization of simpler conditions such as fever was more among poor while the rich reported more consumption of services required for the management of chronic conditions such as kidney problems. [30] The third study showed that in the post insurance period utilization of services in private hospitals increased in Andhra Pradesh and decreased in Maharashtra. On the other hand, utilization in public facilities reduced in both the states with more decrease seen in the state of Andhra Pradesh. [32]

Increase in the utilization rate in early years of implementation was much higher (12.3% to 244%) [26, 35, 36], than the increase in utilization reported (15%) when the scheme was evaluated after 5 years of its implementation. [37]

#### Impact on health

A single study assessed the impact of health insurance on the improvement of health among those enrolled in the scheme. It reported that the mortality rate from conditions covered by the scheme was less in eligible households as compared to ineligible households (0.32% vs 0.90%). [36] While about half (52%) of deaths among enrolled households were among people aged <60 years, this rose to more than three-fourths (76%) among those not enrolled. The study also showed that impact of the scheme in reducing mortality was more pronounced among poor in the treatment areas and not among population above poverty line.

### Process evaluation

Out of the 29 studies without a control group, 77% of them (n = 24) were on RSBY only and the remaining studies either assessed state sponsored health insurance scheme only or compared it with RSBY. The process indicators included in these studies were level of awareness, determinants of enrolment and utilisation and accessibility to hospitals.

Eight studies done across states in India measured the awareness level of various attributes related to the health insurances schemes. [26, 29, 38, 41, 43, 44, 53, 63] Further, 10 studies also assessed the source of awareness about these schemes across various states in India. [26, 29, 38, 41, 43, 44, 53, 57, 63, 66] Furthermore, 6 studies evaluated the role of determinants for enrolment. [37, 42, 46, 47, 49, 50] Similarly, 8 studies measured the association of factors influencing utilization of health services, among the enrolled households. [24, 33, 37, 40, 47–49, 51]

#### Awareness

Awareness levels of various attributes related the insurance schemes were reported to be in the range of 13.6% to 90% as shown in Table 3. Awareness was highest for information on BPL status and 5 member per household as the eligibility criteria and relatively lowest for transport allowances and diseases/conditions covered under the insurance schemes. Specifically, information on eligibility condition of 5 members per household varied from 31% in Chhattisgarh to around 63% in Haryana. Further, awareness level ranged from 32% in Gujarat to 65% in Himachal Pradesh regarding information on free treatment being given under the scheme. Similarly regarding knowledge of transport allowance, information levels ranged from 13.6% in Haryana to 43% in Uttar Pradesh. Panchayats (median: 61%) and friends/ neighbours (median: 44.5%) were the most common source of awareness. In around 60% and 43% of the reported studies, panchayat and friends/neighbour respectively were stated as the source of awareness in more than 60% of the studied population. Less than 15% of the population stated the contribution of health care workers for awareness generation (Table 4).

**Table 3:** Awareness about publicly sponsored health insurance schemes in Indian States.

	Awareness levels among enrolled households						
Domains of Awareness	Chhattisgarh [41, 63]	Gujarat [38, 44]	Haryana [66]	Uttar Pradesh [29]	Himachal Pradesh [43]	Karnataka [26]	Maharashtra [53]
**5 member per family eligibility criteria**	31%	57.3%	63.4%		55%	36.5%	
**BPL status as eligibility criteria**	53.6%-59.6%	47%					
**Free treatment being given**		32.2% -53%	49%		65%		
**Transport allowances**		25%-33.7%	13.6%	43%			17%
**Diseases/conditions covered**				16%	28%		
**Post discharge medication**				53%			
**Empanelled hospitals**					29%	36.5%	
**Limit of hospitalization**	90%						33.6%

**Table 4:** Source of awareness on publicly sponsored health insurance schemes in Indian States.

	Percent Contribution of Awareness Source Among Enrolled Household							
Sources of awareness	Gujarat [38, 44]	Chhattisgarh [41, 63]	Maharashtra [53]	Uttar Pradesh [29]	Himachal Pradesh [43]	Karnataka [26]	Delhi [57]	Haryana [66]
**Panchayat**	46%-85%	34%-75%		14%	61%	80%		
**Friends/Neighbourhood/family member**	10%-21.6%		44%	60%	9%		69%	69%
**Community health workers**	14.6%		0.3%					
**Advertisements**	5%		2%		9%			3%

#### Determinants of enrolment

The studies selected in the review showed that enrolment was inversely associated with administrative areas having a larger geographic size [42, 49] and families belonging to socially disadvantaged communities [42, 46, 50] (Table 5). Further, 2 studies also reported that low enrolment was related to the poverty status of the households. [46, 47] On the contrary, higher enrolment was associated with households headed by a female. [37, 46] Further, districts with good development indicators in terms of better business index [49], low corruption index [46], higher coverage of preventive health services such as DPT immunization [50] and better accessibility to commercial banks or nearby town [50] were also positively associated with high enrolment rates. None of the selected studies identified ‘self-selection’ while analysing the determinants of enrolment although one study mentioned that there is less likelihood of self-selection in RSBY as the scheme is open only for poor. [50]

**Table 5:** Factors associated with enrolment in publicly financed insurance schemes in India.

	Determinants of Enrolment				
Studies	Socio-economically backward groups	Poorest households	Female headed households	Geographical size of the administrative unit	Districts with good development indicators
**Rathi et al. [42]**	Negatively associated			Negatively associated	Positively associated
**Nandi et al. [46]**	Negatively associated	Negatively associated	Positively associated		
**Sun et al. [50]**	Negatively associated				Positively associated
**Narayana et al. [47]**		Negatively associated			
**Ghosh et al. [37]**		Positively associated	Positively associated		
**Das et al. [52]**		Positively associated			
**Krishnaswamy et al. [49]**				Negatively associated	Positively associated

#### Determinants of utilization

**Fig 2:**
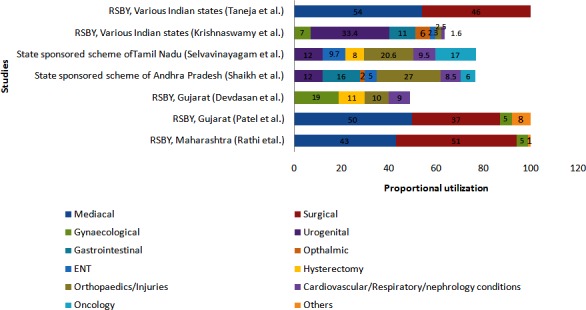
Procedure/speciality-wise utilization under publicly financed health insurance schemes in Indian states.

Higher the number of empanelled hospitals and proportion of private hospitals in a district, higher were the rates of hospitalization [47–49, 51] (Table 6). Less advantaged castes were associated with lowest utilization rates. [24, 33, 37, 40] In contrast to trends in enrolment, districts with better indicators of economic development such as access to educational, commercial, hospitals and transportation institutions and better coverage of preventive or primary health services (such as DPT3 immunization rate) were linked with low utilization rates. [48, 49] RSBY scheme was mostly utilized for gynaecological procedures (5–20%), urogenital (33.4%), gastrointestinal (11%) and ophthalmic (6%) conditions (Fig 2). On the contrary, state sponsored health insurance schemes catered mainly to tertiary care needs for injuries (21–27%), oncology (6–17%) and cardiovascular/respiratory/nephrology conditions (9–10%). RSBY scheme was used predominantly for medical as compared to surgical procedures.

**Table 6:** Factors associated with utilization in publicly financed insurance schemes in India.

	Factors associated with Utilization				
Studies	Socio-economically backward groups	Poorest households	Districts with good development indicators	Total number of empanelled hospitals	Proportion of private empanelled hospitals
**Hou et al. [48]**			Negative association	Positively associated	Positively associated
**Shoree et al. [51]**				Positively associated	Positively associated
**Krishnaswamy et al. [49]**			Negative association	Positively associated	Positively associated
**Narayana et al. [47]**				Positively associated	Positively associated
**Ghosh et al. [37]**	Negatively associated	Negatively associated			
**Devadasan et al. [40]**	Negatively associated				

Private facilities were observed as the preferred ones by the beneficiaries of both RSBY and state level health insurance schemes. Findings from the states of Gujarat [40], Uttar Pradesh [29] and Haryana [66], showed private facilities to be most commonly utilized (73%, 87% and 67% respectively) under RSBY. Three-quarters of all claims under RSBY in India were reported to have utilized care in private facilities, with Bihar, Madhya Pradesh, and Rajasthan reporting 100% of claims from private facilities. [51] Over time, claims in Chattisgarh increased by 266% (INR 38436 to 140900) in private hospitals, as compared to 204% increase in public facilities (INR 30525 to 92905). [45] Considering, state sponsored scheme of Andhra Pradesh, number of surgeries performed in private hospitals were 2.85 times higher than in public facilities. [60]

**Fig 3:**
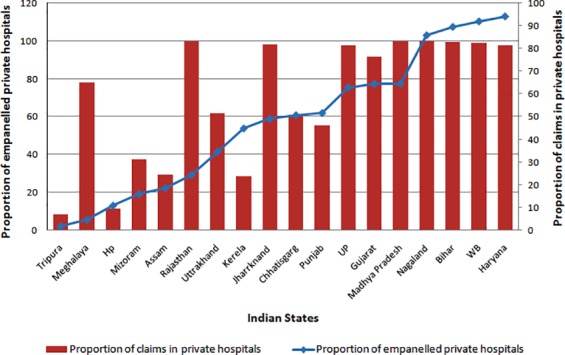
Correlation between private sector claims and density of private empanelled hospitals in the states across India.

It could be assumed that large percentage of empanelled private providers is the reason for high utilization of these facilities under RSBY. The states of Haryana, West Bengal and Bihar, where proportion of private empanelled hospitals was around 90%, the proportion of overall claims in these facilities was more than 95% in each of these states. (Fig 3), [67] Similarly, in Tripura, Himachal Pradesh and Assam where proportion of private facilities was less than 20%, the proportion of claims in these facilities was less than 30%. Districts such as *Kanpur Nagar* from UP, *Dangs* from Gujarat and *Karnal* from Haryana, having more than 90% of total empanelled hospitals as private had highest hospitalisation rate across the state. [47]

Even states with lower private sector empanelment, also continue to show higher share of private sector utilization. Private sector contributed 65% and 25% of the total empanelled facilities in the states of Madhya Pradesh and Rajasthan, while 100% of the claims were from private sector in these states (Fig 3). Similarly, Uttar Pradesh and Jharkhand having 98% of claims from private facilities had 62% and 54% of the total empanelled facilities as private respectively. Kerala and Assam were the outliers, where the despite a proportion of private empanelled hospitals of around 50%, the utilization of these facilities was below 30%.

#### Uniformity and accessibility of hospitals

Hospitalisation rates under RSBY scheme fell steadily with distance of home from health facility. [40] Those who lived more than 30 km had a lower inpatient rates as compared to those who lived within 30 km. Likewise, for Andhra Pradesh’s RAS scheme, as distance from the nearest treatment facility increased, the utilization rates declined. [58] Density of the empanelled hospitals was significantly and positively correlated with the utilization rate. [47, 48]

## Discussion

Historically, the health system in India has had a maternal and child health (MCH) centric approach, both in financing and delivery of health services. [68] Low public spending on health care shifted the burden of seeking care on households by paying out of pocket expenditures. [9] This led to either a barrier in accessing health services, or catastrophic outcomes for those who sought care. [4, 5, 7] Further, low capacity of public health system has resulted in rapid development of private health care delivery system, as well as a push towards various demand-side financing mechanisms. [69, 70] The recent policy thrust on UHC has shifted attention towards a broader focus on health system to meet all the needed preventive as well as curative health care needs of the population.

It is in this contextual framework that various publicly financed health insurance schemes evolved in India. At a time when the debate of ‘how’ to achieve universal health care is raging wide discussions, our paper attempts at summarizing the existing evidence. Our review is the first comprehensive systematic review which focuses on Indian publicly financed health insurance schemes. We find that there is positive evidence that the utilization of hospital services increased after introduction of these insurance schemes. Moreover, this increase in utilization has sustained over time and across regions. However, commensurate with an increase in utilization of services, so far we do not find substantial evidence on reduction of out-of-pocket expenditures or improvement of financial risk protection. In fact, 5 out of 8 studies actually reported either no impact or an increase in OOP expenditures. Finally, although one study does point to some beneficial effect on health of population, there is dearth of robust evidence on the impact of these schemes on the health of the population.

Although our review finds a general increase in utilization of hospitalization services, there are still several unanswered questions. This increase in utilization of hospitalizations could be attributed to 3 reasons: firstly, it could be a result of a pent-up demand on account of previously present barriers to access. However, this could explain the increase in hospitalization during early years of the implementation of health insurance schemes. Persistence of increased utilization over the last 7–8 years rules out this reason. Secondly, it could be attributed to either genuine reduction of financial barriers to access or a supplier induced demand. Given the available evidence, it is difficult to single out the reason from amongst the latter two. Examination of presence and extent of supplier-induced demand is certainly an important future area of research for health economists, although establishing a causal link is fraught with several methodological issues and problems with data availability. It can also be seen that the positive impact on utilization of services which we find in most existing studies could be an underestimate of the true effect considering low awareness level among the enrolled population. As time passes and awareness level improve, this could lead to further increase in utilization of health services [71–73]. Moreover, our review also shows that this increase in utilization is more concentrated in private sector hospitals. Together these two findings imply that it is not only likely to impose fiscal constraints on the government for sustainability of these schemes, but also expected to divert large amount of tax based public money towards private sector.

A second point of concern which points to inefficiency is the presence of conditions such as gynaecological problems, deliveries, cataract etc. among some of leading conditions for which hospitalizations are done. [40, 49] This is a pointer to inefficient allocation of resources since while on one hand the Government is already allocating significant supply-side resources through flagship health programs on strengthening public sector facilities for providing universal access to these conditions [74]; on other hand these conditions continue to be major sources of utilization in the demand-side financing schemes. Considering that much of this utilization in these demand-side financing schemes happens in the private sector, it is inefficient as it leads to double allocation for meeting the same demand. Moreover, this also points to a possible gaming by providers [75, 76], where dual practice could possibly result in siphoning off of public sector demand to private sector for provisioning under these schemes.

Contradicting findings in terms of increase in utilization and lack of significant improvement in financial risk protection needs careful examination. This could be explained based on several possible reasons, Firstly, the height of benefit package under existing schemes such as RSBY is inadequate. With a cover of INR 30,000 per year per household, several high cost illnesses leave the individuals at risk of impoverishment. Secondly, the depth of coverage could possibly be inadequate. RSBY and other state health insurance schemes primarily cover the services requiring hospitalization, while nearly 70% of overall health expenditure is on account of outpatient care which is not covered. [77] So, even enrolled households continue to pay for outpatient care. Thirdly, there is a possibility that even the private empanelled hospitals are charging the patients who pay the same out-of-pocket. [40] Finally, and importantly, it is possible that the bulk of private empanelled providers which exist in the urban areas remain elusive to the vast rural population which continues to face geographic barriers to accessing care. [78] This possibility is also substantiated by the finding that the benefits are mostly gained by the richer quintiles and urban population. In view of limitations of existing evidence, a conclusive statement will require further research which examines these possible explanations. Important policy inferences emerge from the latter point–firstly, that no such demand-side health financing scheme can succeed in providing financial risk protection in the absence of a strong primary health infrastructure. Secondly, this primary health infrastructure needs to be equitably distributed and utilized. Finally, since the rural and disadvantaged areas have not seen the growth of private sector, there is significant merit in the role of investing to strengthen public sector infrastructure.

An important finding from the process evaluation reports is the inequitable nature of the enrolment and utilization. This point towards inefficient targeting towards those who need the services most. Several reasons could be considered to explain this finding. Firstly, insurance companies have an incentive to enrol less than the maximum number of 5 household members, because the premium payment is linked to the number of households enrolled, rather than members. Moreover, villages with higher proportion of BPL population have poorer enrolment. This could be a result of systematic attempt to enrol the better-offs rather than worse offs. Average family size reported in India is 4.8. However studies from the review shows average family size of households under RSBY in the range of 1.46–3.77. [27, 29, 48, 50]. This points to the need for comparing the characteristics of family member enrolled in RSBY against those who are left out. This would help ascertain whether there is any cream skimming by insurance companies. Secondly, it could be seen that in more backward villages, due to paucity of means, poorer households are not able to get a BPL card. And since the means test to identify a poor household is the BPL card, hence the very poor are unable to enrol in the scheme. [42, 47] This in turn could lead to poor targeting under the scheme as most needy and poor are unable to obtain BPL card. Another reason which could contribute to poor enrolment among the poorest could be low level of awareness regarding the means to get an insurance card. This also correlates with the finding of low awareness about publicly sponsored health insurance schemes among the target population. [26, 29, 38, 43, 53]

We would like to acknowledge that impact evaluation was the primary objective of the present paper, and as a result we might have missed out on some studies which were purely describing the processes. Secondly, we are also likely to miss qualitative narrative of the implementation of these insurance programs, and which do provide important insights. This also explains our reporting of impact assessment results first, followed by process evaluation. However, it is also important to understand that the process evaluations in literature are not as standardized as the impact evaluations, which makes it difficult to systematically report. Not every process evaluation reported findings on the same set of indicators. This is an important gap in literature and needs to be bridged in future studies.

## Conclusion

Given the current policy directions for universal health care, publicly financed health insurance schemes are likely to stay. Hence there is a need to design the schemes and implement safeguards so that the benefits of the risk pooling can be maximized. Firstly, benefits of these demand-side financing mechanisms will be not reaped unless the basic health care infrastructure for delivery of primary health services is strong. This primary health care infrastructure will be necessary to provide basic health services, besides serving as gatekeeping for specialist services. Examples from Thailand, United Kingdom and Mexico substantiate this claim. [79] Secondly, the public sector needs to be strengthened and incentivized to compete for provision of services. This will generate much needed extra revenue for the public health system, which can in turn be used to strengthen provision of health services. The public sector has demonstrated that it can provide universal access for health care services, which are delivered efficiently and utilized equitably, the only condition being that enough resources are spent. Various interventions for improving access to maternal health care services and institutional delivery in public sector illustrates this point. [80–82] Thirdly, there is a need to invest in systems to monitor and evaluate implementation of health insurance schemes. This is also essential in view of large private sector presence, which has perverse incentives to induce demand; and the intermediary purchaser/ insurer, who has perverse incentive to reduce utilization through cream-skimming. Overall, publicly financed health insurance schemes are not the panacea to achieve UHC in India. Instead, these schemes need to be aligned with proper strengthening of the public sector for provision of comprehensive primary health care. Secondly, presence of health insurance schemes could be used as an opportunity to reform the tenets of the health sector which are beyond the routine regulatory frameworks.

## Supporting Information

S1 Table PRISMA Checklist. (DOC)
